# Supplementary figures and images for: Intrinsic growth heterogeneity of mouse leukemia cells underlies differential susceptibility to a growth-inhibiting anticancer drug
Source: PLoS One. 2021 Feb 1;16(2):e0236534. doi: 10.1371/journal.pone.0236534 (PMC7850478; doi:10.1371/journal.pone.0236534)

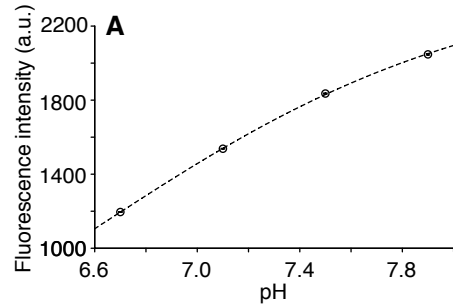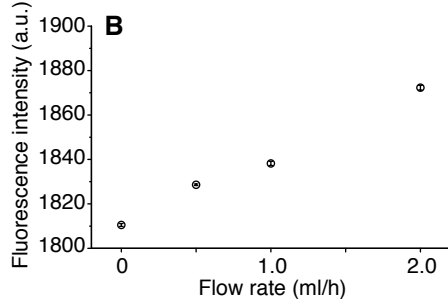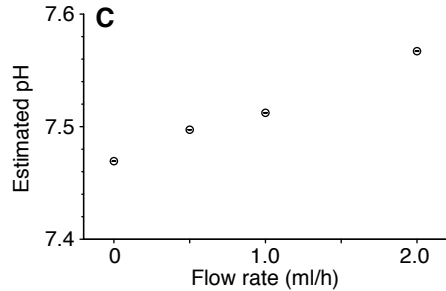

Supplement: S1 Fig — (A) Fluorescein fluorescence is dependent on pH. 0.1 mM fluorescein solution buffered with 100 mM HEPES (pH 6.7, 7.1, 7.5, or 7.9) was introduced into the device, and fluorescence images in the trench were acquired by microscopy. The points show the measured relationship between the pH of the flowing solution and the fluorescence intensity. The error bars, which are smaller than the points, were the standard deviation of the fluorescent intensity of the images acquired at five times with a 1-min interval at the same position in the trench channel. The broken curve represents the fitting of the data points by a Hill function y=ymaxxnxn+Cn with the parameters ymax = (2.53±0.05) × 103 a.u., C = 6.78±0.03, and n = 9.5±0.3. (B) pH of the culture medium in the microfluidic device is robust to fluctuation in the medium flow rate. RPMI-1640 medium containing 0.1 mM fluorescein as a pH reporter was introduced into the device under several conditions of flow rate. The device was placed in the 5% CO2 atmosphere on the microscope stage, and fluorescence images were acquired. The plot shows the relationship between the flow rate of RPMI-1640 medium and fluorescence intensity. (C) Relationship between flow rate and pH of RPMI-1640 medium estimated from the results in (A) and (B). (PDF) [file pone.0236534.s003.pdf]

**A**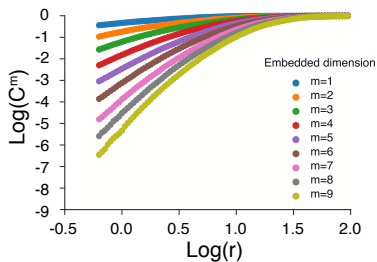**B**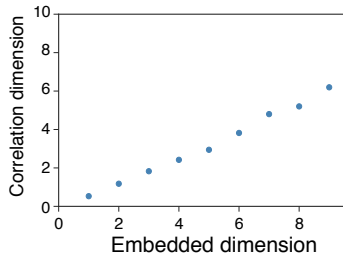**C**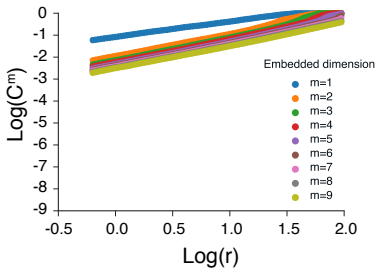**D**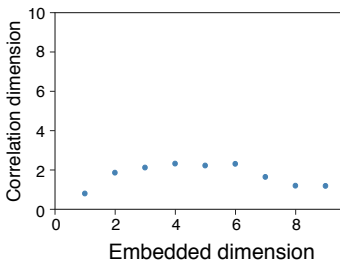**E**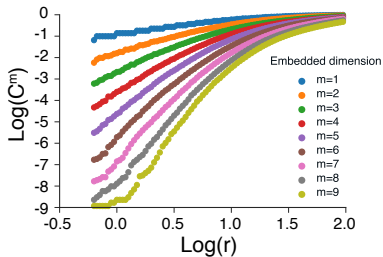**F**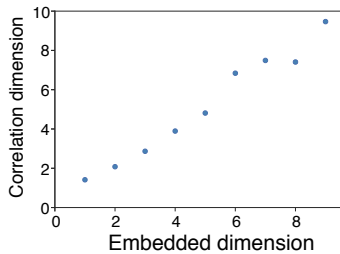

Supplement: S2 Fig — (A and B) An example of correlation dimension analysis for a stochastic process. Generation time series data were generated by random sampling from a shifted-gamma distribution and embedded in a m-dimensional space (m = 1 to 9). Correlation integral Cm(r) was calculated for various r and plotted on a log-log scale (A). Correlation dimension, which is a slope of the logCm v.s. logr curve in the intermediate ranges of r where the slope is nearly constant, increased with the embedded dimension, m. This is a typical consequence for a stochastic process (B). (C and D) The same analyses as (A) and (B) except that generation time series were simulated according to a deterministic cell cycle model of L1210 [28]. Unlike stochastic processes, correlation dimension converged to a certain value with increase in m (D). (E and F) Correlation dimension analysis for experimental data including 301 cell lineages of more than 11 generations. The results were qualitatively similar to (A) and (B), suggesting a stochastic nature of L1210 cell cycle dynamics in our system. (PDF) [file pone.0236534.s004.pdf]

**A**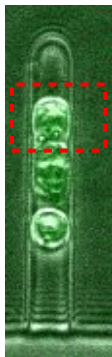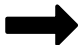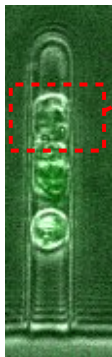

Collapsed  
cell morphology

**B**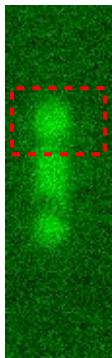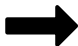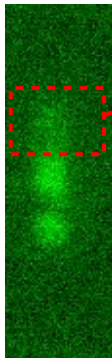

Loss of  
fluorescence  
signals

Supplement: S3 Fig — (A) Microscopic images of a cell exhibiting death in a growth channel. An intact-looking cell in the red rectangular collapsed its cell morphology by the next time point in the time-lapse measurement. Since these collapsed cells stopped moving and never regrew, we judged them dead. (B) The fluorescence images of a cell. The images correspond to those in A. The mVenus signal in the cell dropped significantly by the next time point. Since these cells stopped moving and never regrew, we also regarded the loss of mVenus fluorescence signals as the indication of cell death. (PDF) [file pone.0236534.s005.pdf]
